# The application of decision tree analysis based on nursing vertical management in the allocation of obstetrics and gynecology nurses

**DOI:** 10.4314/ahs.v25i2.29

**Published:** 2025-06

**Authors:** Xia Wu, Yu Liu

**Affiliations:** Department of Gynecology, Second Hospital of Tianjin Medical University, Tianjin, China

**Keywords:** Nursing vertical management, Decision tree analysis method, Nurse allocation, Obstetrics and gynecology

## Abstract

**Background:**

Addressing nursing resource challenges in obstetrics and gynecology, this study employs decision tree analysis within nursing vertical management to enhance allocation efficiency and patient care. This study aimed to assess the efficacy of employing decision tree analysis within nursing vertical management for allocating obstetrics and gynecology nurses.

**Methodology:**

Sixty-one obstetrics and gynecology nurses were included in the study from January to December 2022 at our hospital. The control group, from January to June 2022, underwent routine nurse allocation management, while the observation group, from July to December 2022, experienced nurse allocation based on nursing vertical management using decision tree analysis. Nursing quality scores and satisfaction with allocation were statistically compared between the groups. Additionally, obstetrics and gynecology nursing level, illness matching rate, nursing quality scores, and patient satisfaction were evaluated.

**Results:**

The observation group showed significantly higher nursing quality scores and greater satisfaction with allocation compared to the control group. Moreover, the observation group exhibited higher obstetrics and gynecology nursing levels, illness matching rates, nursing quality scores, and patient satisfaction compared to the control group, all with statistical significance (P<0.05).

**Conclusions:**

Implementing the decision tree analysis method within nursing vertical management notably enhanced both satisfaction levels and the quality of care among obstetrics and gynecology nurses. It outperformed traditional deployment management, ensuring improved nursing work quality and heightened patient satisfaction. This approach is pivotal for fostering harmonious nurse-patient relationships and deserves widespread adoption.

## Introduction

Recently, the incidence rates of various diseases have been increasing year by year. Obstetrics and gynecology nursing in clinical practice involves high technical risks, demanding requirements, and a heavy workload. Consequently, adverse events often occur and develop in clinical settings. However, there is a relative shortage   of clinical nursing staff in our country. Allocating nurses reasonably and improving work efficiency has become a significant concern in clinical practice. The decision tree is a widely used data mining technique with precise classification capabilities. Decision tree analysis offers a systematic approach to decision-making, enabling clear visualization of choices and their potential outcomes. This method is particularly effective in scenarios with multiple influencing factors and possible solutions, like nurse allocation. It helps in identifying the most efficient pathways based on various criteria, such as nurse qualifications, patient needs, and workload distribution. In the field of medical research, it mainly focuses on genomics-related studies and classification, attracting widespread attention and development. Nursing vertical management is a nursing management model that has been widely promoted and applied in clinical practice in recent years. Some scholars have highlighted that adopting the vertical nursing management model can effectively enhance work efficiency and nursing quality, while avoiding constraints from non-professional management^1^-^2^. By integrating these two management techniques, with nursing vertical management as the foundation and utilizing the decision tree analysis method for allocation management based on current clinical situations, managers can objectively allocate nurses, achieve better allocation efficiency, and ensure the smooth implementation of clinical nursing work^3^-^4^. The primary objective of this study is to evaluate the effectiveness of integrating decision tree analysis within nursing vertical management for the allocation of nursing resources in obstetrics and gynecology departments. This research aims to compare this innovative approach with traditional hierarchical management methods, focusing on outcomes such as nurse care quality scores and job satisfaction. By doing so, the study seeks to provide empirical evidence on the potential benefits and practical implications of this approach, guiding healthcare institutions in optimizing nursing resource allocation and enhancing overall patient care

## Materials and Methods

### Baseline Information

A total of 61 obstetrics and gynecology nurses were enrolled in our study at our hospital from January 2022 to December 2022. From January 2022 to June 2022, routine nurse allocation management was divided into the control group, and from July 2022 to December 2022, nurse allocation based on nursing vertical management using the decision tree analysis method was divided into the observation group. The members of both groups were from the same batch, including 17 outpatient nurses and 44 ward nurses, aged between 24 and 51 years old. Among them, 58 had a bachelor degree or higher, and 3 had an associate degree. The working experience ranged from 2 to 32 years. There were 83 available beds, 4 deputy chief nurses, 17 head nurses, 35 staff nurses, and 5 nurses in total.

### Methods

**Control Group:** The control group utilized a traditional management model, implementing the following strategies: 1) The hierarchical management system was established following traditional models of nursing administration. This system was structured based on seniority and educational qualifications, where nurses were assigned to roles and responsibilities primarily according to their experience levels and academic credentials. The process began with an assessment of the department's overall nursing needs, taking into account patient volumes, complexity of cases, and required nurse-patient ratios. 2) A tiered structure was developed. At the base level, newly graduated or less experienced nurses were assigned to general care tasks, under the supervision of more senior nurses. Mid-level nurses, possessing greater experience or specialized qualifications, were tasked with more complex care responsibilities and some administrative duties. The top tier consisted of highly experienced nurses, responsible for overseeing clinical operations, mentoring junior staff, and directly handling the most complex cases. Nurses were classified into three levels - junior, intermediate, and senior - considering their working hours, abilities, and professional titles. Junior nurses focused on enhancing their professional skills and basic nursing practices, primarily responsible for caring for patients with less severe conditions. Intermediate nurses engaged in clinical learning, teaching, and ward rounds, assuming the care of more critically ill patients. Senior nurses assisted nurse managers in clinical research and ward management, being responsible for critically ill patients' care. 3) A nurse human resource pool was established to strengthen the development of nurse talent progression. Nurses from different levels were allocated and trained based on their specific requirements. Training programs included professional learning, lectures, ward rounds, and daily proactive training. Higher-level nurses delegated training responsibilities to lower-level nurses to enhance motivation. Experienced nurses facilitated mutual learning and supervision among nurses at different levels. Outstanding nurses were selected for further education and training, combining internal and external opportunities. 4) Nurse performance evaluation was integrated with hierarchical management, establishing a scientific salary distribution system. Performance coefficients were assigned to nurses at different levels, considering patient satisfaction, nursing service quality, technical requirements, job responsibilities, and risk levels. Promotions and recognition correlated with performance evaluation results, with a focus on positions with higher risks, technical requirements, and nursing workloads. This eflected the principle of rewarding exceptional performance and additional workload.

**Observation Group:** The observation group implemented nurse allocation based on nursing vertical management using the decision tree analysis method. The following approaches were employed: 1) Nursing workload and the average nurse-to-patient ratio in the obstetrics and gynecology ward were used as variables. Relevant data related to workload indicators were entered into an information system. The information system generated real-time statistics of unit nursing workload for a specified time period. 2) SPSS 20.0 was used for decision tree analysis, employing the chi-square automatic interaction detection method. The average nurse-to-patient ratio was set as the dependent variable, while nursing days and the number of admitted patients were set as independent variables. The analysis was conducted using the automatic interaction detection method to comprehensively evaluate nursing workload and human resources in the obstetrics and gynecology department. Based on actual workload and resource allocation, a reassignment of nurse workload clusters at different levels in the ward was determined.

### Observational Indicators

Nursing quality scores were collected for both groups, including scores for specialized nursing, basic nursing, disinfection and isolation, nursing safety, health education, and nursing documentation. Each dimension was scored on a scale of 100 points, with higher scores indicating better nursing quality. Higher scores indicate a more effective allocation of nursing resources, leading to improved patient care. Nurse satisfaction was assessed using a hospital nurse satisfaction survey questionnaire. The questionnaire covered aspects such as job assignments, incentive mechanisms, salary benefits, work atmosphere, and personal sense of achievement. Satisfaction levels were categorized as satisfied, neutral, or dissatisfied. Observation items were developed based on actual obstetrics and gynecology patient conditions and the grading nursing guidelines issued by the Ministry of Health. These items included nursing level, patient acuity matching rate, nursing quality scores, and patient satisfaction. Scores were given on a percentage scale, with higher scores indicating higher satisfaction levels.

### Statistical Analysis

Statistic Package for Social Science (SPSS) 25.0 (IBM, Armonk, NY, USA) was used for data analysis. Measurement data were expressed as mean ± standard deviation, and independent sample t-tests were used for intergroup comparisons. Count data were expressed as percentage and analyzed using the Z-test. A statistical significance level of P<0.05 was considered significant.

## Results

### Analysis of the nursing quality scores of two groups

Table 1 displayed a side-by-side comparison of nursing quality scores across various categories between the observation group and the control group, each consisting of 61 nurses. The categories examined include specialized nursing, basic nursing, disinfection and isolation, nursing safety, health education, and nursing documentation writing. The observation group, which was subject to the decision tree analysis and nursing vertical management intervention, consistently outperformed the control group. The mean scores (± standard deviation) for the observation group were significantly higher in all categories: specialized nursing (93.18±2.48), basic nursing (91.27±3.22), disinfection and isolation (94.18±3.25), nursing safety (92.19±3.64), health education (92.19±4.38), and nursing documentation writing (90.19±3.24). In contrast, the control group, which followed a traditional hierarchical management system, scored lower, with mean values of 88.74±3.27 for specialized nursing, 85.19±3.24 for basic nursing, 84.18±3.52 for disinfection and isolation, 87.17±3.24 for nursing safety, 88.28±3.28 for health education, and 86.84±3.75 for nursing documentation writing. The P-values for all categories are 0.001, indicating that the differences in mean scores between the groups are statistically significant. These results suggest that the decision tree analysis within nursing vertical management can effectively enhance the quality of nursing care across various specialized and fundamental nursing tasks.

**Table 1:** Comparative analysis of nursing quality scores between two groups

Group	n	Specialized nursing	Basic nursing	Disinfection and isolation	Nursing safety	Health education	Nursing documentation writing
Observation group	61	93.18±2.48	91.27±3.22	94.18±3.25	92.19±3.64	92.19±4.38	90.19±3.24
Control group	61	88.74±3.27	85.19±3.24	84.18±3.52	87.17±3.24	88.28±3.28	86.84±3.75
*t*	--	8.325	4.698	16.302	8.046	5.581	5.280
*P*	--	0.001	0.001	0.001	0.001	0.001	0.001

### Analysis of the satisfaction of two groups of nurses regarding job assignments

**Figure 2:**
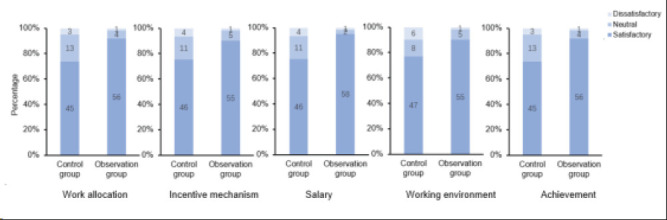
Comparative analysis of job satisfaction regarding nurse deployment between two groups

The satisfaction of nurses in the observation group with their allocation was higher than that in the control group, with statistical significance (P<0.05), as shown in Table 2 and Figure 2.

**Table 2:** Comparative analysis of job satisfaction regarding nurse deployment between two groups

n		Observation group	Control group	*Z*	*P*
	Satisfactory	56(91.8)	45(73.8)		
Work allocation	Neutral	4(6.6)	13(21.3)	31.274	0.001
	Dissatisfactory	1(1.6)	3(4.9)		
	Satisfactory	55(90.2)	46(75.4)		
Incentive mechanism	Neutral	5(8.2)	11(18.0)	36.183	0.001
	Dissatisfactory	1(1.6)	4(6.6)		
	Satisfactory	58(95.1)	46(75.4)		
Salary	Neutral	2(3.3)	11(18.0)	33.183	0.001
	Dissatisfactory	1(1.6)	4(6.6)		
	Satisfactory	55(90.2)	47(77.0)		
Working environment	Neutral	5(8.2)	8(13.1)	34.298	0.001
	Dissatisfactory	1(1.6)	6(9.9)		
	Satisfactory	56(91.8)	45(73.8)		
Achievement	Neutral	4(6.6)	13(21.3)	31.984	0.001
	Dissatisfactory	1(1.6)	3(4.9)		

### Comparative analysis of two groups' obstetrics and gynecology nursing levels, condition matching rates, nursing quality scores, and patient satisfaction

**Figure 3:**
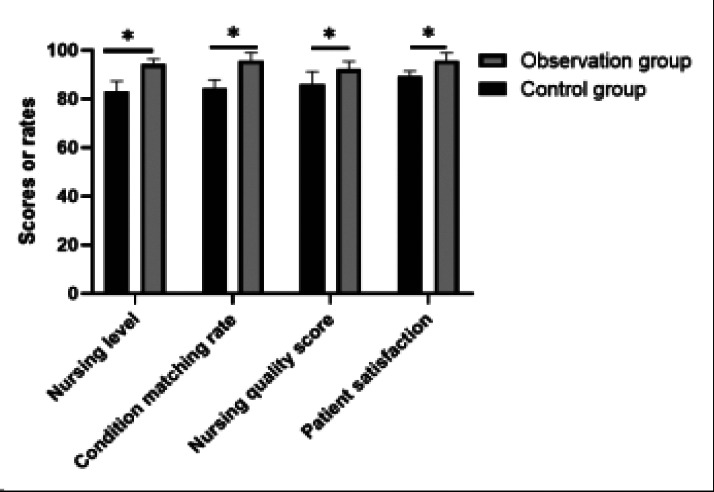
Comparative analysis of two groups' obstetrics and gynecology nursing levels, condition matching rates, nursing quality scores, and patient satisfaction. *P <0.05

The observation group had higher levels of obstetric and gynecologic nursing, matching of patient conditions, nurse care quality score, and patient satisfaction compared to the control group, with a statistical significance (P<0.05), as shown in Table 3 and Figure 3.

**Table 3:** Comparative analysis of two groups' obstetrics and gynecology nursing levels, condition matching rates, nursing quality scores, and patient satisfaction

Group	n	Nursing level	Condition matching rate	Nursing quality score	Patient satisfaction
Observation group	61	94.18±2.18	95.82±3.28	92.18±3.25	95.82±3.24
Control group	61	83.18±4.18	84.29±3.64	85.84±5.46	89.18±2.24
*t*	--	18.224	18.219	7.793	13.16
*P*	--	0.001	0.001	0.001	0.001

## Discussion

Gynecology and obstetrics is one of the four major departments in clinical medicine, mainly treating a large number of gynecological diseases and pregnant women. It is also the primary location for treating gynecological and obstetric diseases. Currently, influenced by various factors, the patient flow in hospitals is increasing day by day, and the public's demands for clinical gynecological and obstetric care are constantly rising. Nursing staff face significant technical risks and high work pressure. In this context, adverse events often occur in nursing, which is very detrimental to the physical and mental health of nursing personnel and the nurse-patient relationship^5^-^6^. Nursing-related adverse events can gravely affect both nurses and patients. For nurses, these events may lead to stress, job dissatisfaction, and a sense of failure, heightening the risk of burnout. In terms of the nurse-patient relationship, trust can be compromised, potentially decreasing patient satisfaction and engagement in their care.

Furthermore, such events can prolong hospital stays and inflate healthcare costs. Preventative measures are essential, involving adequate staffing, continuous training, and adherence to best practices to maintain high-quality care and ensure the well-being of both nursing staff and patients. In addition, at present, there is a shortage of nursing resources in clinical settings in China, and all departments face situations of inadequate manpower. The medical resources in the field of gynecology and obstetrics are also gradually becoming tense. How to improve the efficiency of gynecological and obstetric nursing has become a key focus in clinical care. Some research suggests that to effectively improve the quality of gynecological and obstetric nursing management, nursing managers should scientifically allocate and intervene in the nursing manpower management model based on the actual working conditions of the department. They should strive to ensure the reasonable allocation of gynecological and obstetric nurses, maximize nursing efficiency within available manpower resources, ensure the nursing effects on patients, and alleviate the current shortage of gynecological and obstetric personnel. To achieve this nursing goal, clinical nursing managers need to rationally allocate nursing resources^7^-^9^. However, due to various factors, there is a widespread problem of irrational allocation of nursing positions in clinical settings in China, and the shortage of nursing personnel information is particularly prominent. Unclear job responsibilities hinder the improvement of nursing management quality, becoming a major factor restricting the enhancement of management quality^10^-^12^. In China's obstetrics and gynecology departments, the scarcity of nursing resources exacerbates challenges such as heavy workloads, stress, and diminished patient care quality. With too few nurses for a growing patient population, work pressures increase, leading to potential job dissatisfaction and higher turnover rates. This shortage also hinders the quality of nurse-patient relationships, essential in this sensitive care field. Addressing this scarcity through efficient resource allocation, like integrating decision tree analysis, is crucial for enhancing care quality and supporting the well-being of both nurses and patients within the Chinese healthcare system. Therefore, effectively deepening the connotation and extension of high-quality gynecological and obstetric nursing services in clinical practice, optimizing the allocation of nursing human resources, improving the effective utilization rate of nursing tlents, and nursing management efficiency, continuously exploring new nursing management models, and constructing a more professional and scientific nursing human resources management model will contribute to the rational use of nursing human resources and improve clinical impact efficiency.

Nursing vertical management refers to the implementation of nursing management under the leadership of nursing management personnel based on the requirements of modern scientific management and the needs of hierarchical hospital management. This approach can contribute to the rapid development of the nursing industry and provide higher quality nursing services to patients. Nursing vertical management establishes an independent nursing management system, allowing for the independent management of hospital nurses. This includes the formulation of independent management standards, independent training plans, independent budget responsibilities, etc. It can enhance the work capabilities and performance of nursing staff, achieving organizational strategic goals^5^. In recent years, with the continuous transformation of the medical model in China, nursing vertical management has gradually been widely applied in clinical settings. It has proven beneficial in improving the efficiency and quality of nurses' work in various departments, helping to avoid non-professional management constraints. Research indicates that vertical nursing management can help promote the overall development of nursing science, enhance nurses' professional abilities, and minimize conflicts between nurses and patients, playing a crucial role in improving the currently tense nurse-patient relationship.

In the deployment of obstetric and gynecological nurses, nursing vertical management, through its model, effectively implements personnel management through systems, retains personnel through incentives, and employs personnel through standardized processes. By strengthening institutional constraints and clarifying job responsibilities in nursing work, it helps to fully mobilize the initiative and enthusiasm of nursing staff, improving work efficiency and quality^13^-^15^. Additionally, the vertical nursing model, through a three-level management approach, enhances supervision and management at all levels. This shift from a supervisory to a self-discipline management model is conducive to raising the level of nursing management and further improving the initiative and enthusiasm of nurses. It transforms passive management into a participatory model, playing a crucial role in enhancing the overall teamwork and nursing capabilities of nurses.

Decision tree is a data mining technique widely used in various industries for precise classification. In the medical field, it is primarily applied in genomics and classification, achieving good results. However, in recent years, as clinical research deepens, scholars have discovered that decision trees can also be applied to the daily work deployment of nurses. Decision tree analysis can comprehensively analyze and classify the distribution of nursing manpower and workload in departments, providing objective and accurate data^16^. The decision tree analysis method involves branching out each decision or event into two or more events with different outcomes. This graphical analysis aims to improve the quality of management. The decision tree analysis method utilizes probability analysis principles in nurse management, formulating deployment plans for different levels of nurses based on the actual needs of obstetric and gynecological nurse workloads. This helps in obtaining the optimal deployment scheme, improving deployment efficiency, and satisfaction^17^-^18^. By dividing obstetric and gynecological nurses into different clusters and reasonably allocating based on the actual situation of nurses, using decision tree classification analysis combined with nursing vertical management can scientifically deploy nurses and alleviate nursing workload as much as possible, rationalizing the use of nursing personnel resources. In the vertical management of obstetric and gynecological nurse deployment, the application of decision tree classification is mainly based on specific, objective workload data. Mathematical calculations are used for scientific classification analysis, guiding nurse deployment. The parameters considered include calculating nursing workload and distribution within a specified time period based on information provided by the hospital information system. This involves classifying the load clusters of nursing units, providing data for deploying nursing resources, reducing subjective judgments by nursing managers, and relying more on objective data to guide manpower deployment. This helps avoid subjective judgments and effectively increases the acceptance of nursing staff and manageent personnel^19^. The integration of decision tree analysis with nursing vertical management significantly enhances workload management and resource optimization in nursing. Decision tree analysis efficiently allocates nurses by matching their skills with patient needs, ensuring each nurse's tasks align with their expertise. Concurrently, nursing vertical management establishes a clear hierarchy, appropriately engaging nurses at various levels. This synergy not only prevents staff burnout and underutilization but also boosts job satisfaction and patient care quality. Such a combined approach is pivotal for healthcare institutions aiming to balance nursing workloads and respond effectively to the dynamic demands of modern healthcare.

This study investigated the application value of the decision tree analysis method based on nursing vertical management in the deployment of obstetric and gynecological nurses. Research data indicates that the observation group of nurses scored higher in nursing quality, satisfaction with deployment, obstetric and gynecological nursing level, condition matching rate, nursing quality, and patient satisfaction compared to the control group. The study data suggests that applying the decision tree analysis based on nursing vertical management to obstetric and gynecological nurse deployment can help improve nursing management quality and nurse satisfaction with deployment. This is because, in the deployment of obstetric and gynecological nurses, decision tree analysis based on nursing vertical management can allocate resources according to the actual workload and nursing capabilities, ensuring efficient use of personnel and rational distribution of nursing resources. Furthermore, the decision tree analysis method based on nursing vertical management can directly enhance the training management of nurses, contributing to the improvement of nurses' professional abilities and providing higher quality nursing services to patients^13^,^20^. In such a nursing model, patients can perceive the professional competence of nursing staff, fostering trust in them. This plays a crucial role in promoting the harmonious development of nurse-patient relationships. However, it is important to note that the time period for retrieving data from the hospital information system should not be too short. Otherwise, it may lead to distorted analysis results and an inability to accurately and effectively reflect the daily work situation. In summary, the decision tree analysis method based on nursing vertical management can contribute to increased satisfaction and nursing quality in the deployment of obstetric and gynecological nurses. This research underscores the practical implications of integrating decision tree analysis with nursing vertical management, particularly in enhancing nurse-patient relationships and nursing capabilities. By aligning nurse skills more precisely with patient needs, this approach fosters more personalized and effective patient care. Nurses, allocated tasks fitting their expertise, can provide higher quality care, leading to stronger nurse-patient rapport and trust. Moreover, this method allows nurses to develop specialized skills, enhancing their professional capabilities. Consequently, this innovative approach not only elevates the standard of care but also contributes to a more fulfilling and efficient nursing work environment

## Figures and Tables

**Figure 1:**
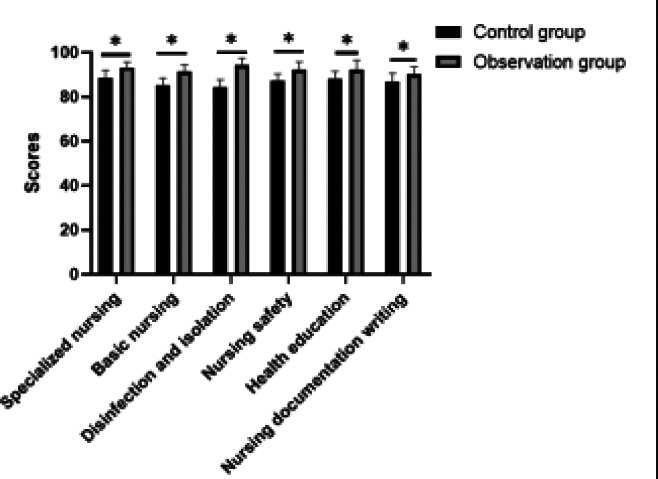
Comparative analysis of nursing quality scores between two groups. * P <0.05
